# A Microshutter for the Nanofabrication of Plasmonic Metal Alloys with Single Nanoparticle Composition Control

**DOI:** 10.1021/acsnano.3c04147

**Published:** 2023-08-03

**Authors:** Carl Andersson, Olga Serebrennikova, Christopher Tiburski, Svetlana Alekseeva, Joachim Fritzsche, Christoph Langhammer

**Affiliations:** † Department of Physics, Chalmers University of Technology, 412 96 Göteborg, Sweden; ‡ ConScience AB, Läraregatan 3, 411 33 Göteborg, Sweden

**Keywords:** microshutter, nanoalloys, nanoparticles, single particle, plasmonic, physical vapor deposition, nanofabrication

## Abstract

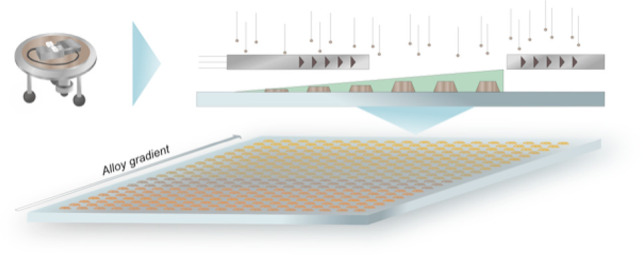

Alloying offers an increasingly important handle in nanomaterials design in addition to the already widely explored size and geometry of nanostructures of interest. As the key trait, the mixing of elements at the atomic level enables nanomaterials with physical or chemical properties that cannot be obtained by a single element alone, and subtle compositional variations can significantly impact these properties. Alongside the great potential of alloying, the experimental scrutiny of its impact on nanomaterial function is a challenge because the parameter space that encompasses nanostructure size, geometry, chemical composition, and structural atomic-level differences among individuals is vast and requires unrealistically large sample sets if statistically relevant and systematic data are to be obtained. To address this challenge, we have developed a microshutter device for spatially highly resolved physical vapor deposition in the lithography-based fabrication of nanostructured surfaces. As we demonstrate, it enables establishing compositional gradients across a surface with single nanostructure resolution in terms of alloy composition, which subsequently can be probed in a single experiment. As a showcase, we have nanofabricated arrays of AuAg, AuPd, and AgPd alloy nanoparticles with compositions systematically controlled at the level of single particle rows, as verified by energy dispersive X-ray and single particle plasmonic nanospectroscopy measurements, which we also compared to finite-difference time-domain simulations. Finally, motivated by their application in state-of-the-art plasmonic hydrogen sensors, we investigated PdAu alloy gradient arrays for their hydrogen sorption properties. We found distinctly composition-dependent kinetics and hysteresis and revealed a composition-dependent contribution of a single nanoparticle response to the ensemble average, which highlights the importance of alloy composition screening in single experiments with single nanoparticle resolution, as offered by the microshutter nanofabrication approach.

Nanostructures and nanoparticles on surfaces are the workhorse of a myriad of nanotechnologies that include electronics,^1,2^ optical metamaterials,^3,4^ sensors,^5,6^ and quantum technologies.^7^ A key contributing factor to their success during the last decades has been the ever-increasing accuracy of size and shape control of the involved nanostructures, where experimental investigations of individual nanoparticles, and the impact of their size and shape on function, is a great example that highlights the level of individuality between particles at the atomic level and thus the importance of accurate control of nanoparticle structure.^8−14^ Importantly, however, this development has to the largest extent taken place on the basis of nanostructures composed of single elements and often metals. At the same time, there are many examples across disciplines where expansion of the material composition space has unlocked new and advantageous properties. Metal alloying, which has played a key role in technology since the Bronze Age, is one of the most prominent examples that enables material properties and functions that cannot be obtained by a single metallic element alone. At the nanoscale, alloying is increasingly used in for instance heterogeneous catalysis to engineer activity and selectivity of surface reactions,^15−17^ as well as in hydrogen sensors,^18^ nano-optics,^17,19,20^ and biological applications like bioimaging or photothermal therapy.^21^

However, while offering fantastic opportunities to truly tailor material properties and functions at the nanoscale, adding the compositional dimension to the structural dimension dramatically increases the level of complexity when investigating nanostructures. The reason is that the parameter space to control becomes very large and almost unmanageable if statistically relevant data sets are to be acquired and traditional experimental methodologies are to be used, since they typically require a dedicated sample for each unique combination of nanoparticle size, shape, and composition. This approach, among several challenges, introduces a high level of experiment-to-experiment uncertainty in the obtained results. Consequently, an attractive solution would be to both nanofabricate and study surfaces composed of arrays of nanostructures with controlled size and shape combined with a compositional gradient tailored at the level of the individual nanostructure.

Focusing first on the aspect of generating compositional gradients in films on surfaces, electrodeposition has been used and some attempts to generate particles in the millimeter and micrometer size range have been reported.^22−26^ Similarly, magnetron sputtering has been applied to grow thin films with compositional gradients on surfaces and to study material function for a large number of compositions on a single sample in a single experiment.^27−30^ To the best of our knowledge, in the cases where nanostructures with compositional gradients have been reported, either (i) co-sputtering or wet-chemical synthesis methods were used, both of which lack a high degree of spatial resolution and reproducibility,^31,32^ or (ii) the compositional gradient was made in such a way that the nanoparticle array had to be spread out over a rather large (millimeter order of magnitude) area.^32,33^ We also note that with co-sputtering it is difficult to control or modify compositional gradients *between* samples since it would entail the repositioning of the sputter sources in the chamber.

Turning to nanostructured surfaces with compositional gradients, a gas aggregation cluster source equipped with a movable mask has been used to generate a Ag- and Cu-nanoparticle surface with a gradient in the local ratio between the two types of single element particles.^34^ Yet a higher level of control can be achieved with physical vapor deposition (PVD) in combination with nanolithography on a surface, which has been successfully used to grow alloy nanoparticle arrays with uniform composition,^35^ single element nanoparticle arrays with a height or spatial orientation gradient,^36^ and single element particles with a geometrical gradient.^37^ However, to the best of our knowledge, no methodology exists that enables the nanofabrication of surfaces in which both nanostructure size and shape are controlled with nanometer resolution and across which compositional gradients can be established with spatial control at the level of the individual nanostructure and with compositional control down to, depending on the materials, 1 at. %. Therefore, in this work, we present a microshutter device that enables the nanofabrication of surfaces with a high level of structural and compositional control in combination with PVD and nanolithography and we exemplify it on arrays of nanodisks with compositional gradients. Second, on the example of the three selected alloy systems AuAg, AuPd, and AgPd, we demonstrate how the obtained surfaces can be used to screen the plasmonic as well as hydrogen sorption properties of up to 3344 nanoparticles in a single experiment and with single particle spatial and compositional resolution.

## Results and Discussion

**Figure 1:**
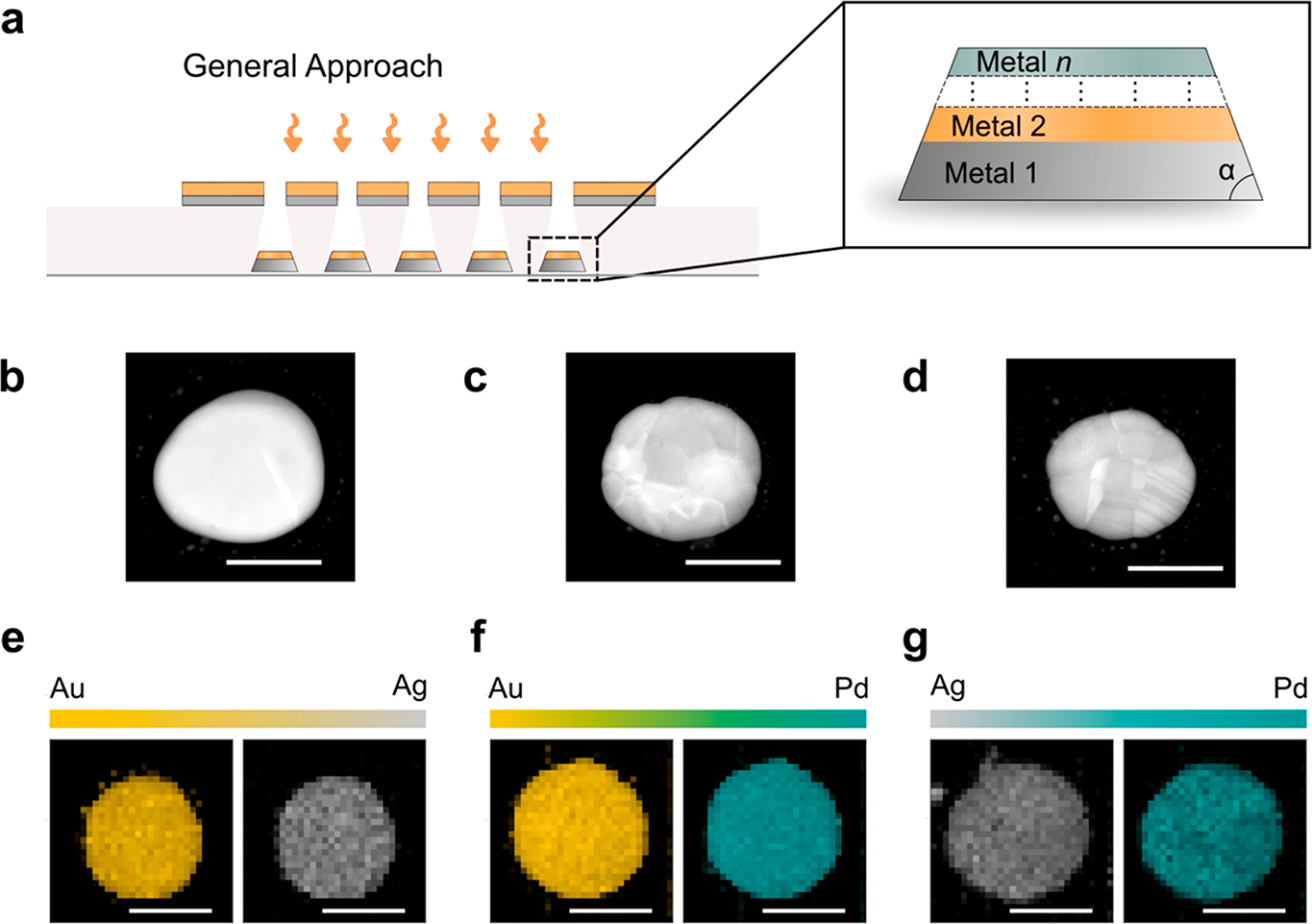
Subsequent deposition of metals in a prefabricated mask and representative, final nanoparticles. (a) Schematic depiction of our general approach for the nanofabrication of alloy nanoparticle using nanolithography.^35^ The desired number of targeted alloy constituents, *n*, is subsequently evaporated through a nanolithography mask. After the lift-off step, the sample is thermally annealed to induce alloy formation between the layers. As the key point, the alloy composition can be accurately controlled via thickness of the evaporated layers. We also note that evaporation through a mask with circular nanometric holes, as in this work, leads to particles that have a truncated cone shape due to a “hole-closing effect” caused by continuous material buildup on the rim and thus closing of the mask during evaporation. Importantly, the slope angle of the truncated cone, α, depends on the evaporated material and is around 60° for the metals used in this work. Representative HAADF-STEM images of 50 wt % AuAg (b), AuPd (c), and AgPd (d) alloy nanoparticles produced in this work, which reveal the particle morphology after annealing. Scale bar: 100 nm. TEM EDS maps of single 50 wt % AuAg (e), AuPd (f), and AgPd (g) nanoparticles on the same samples as (b)–(d), which confirm the homogeneous distribution of the constituents in all three alloy systems. Scale bar = 100 nm.

The general foundation of our approach is the subsequent PVD of thin metal films of different metallic elements through a supported, prefabricated nanolithography mask (Figure 1a), which after lift-off and thermal annealing of the nanofabricated surface above the recrystallization temperature of the system at hand generates arrays of homogeneously alloyed nanoparticles on the surface, as we earlier have demonstrated using hole-mask colloidal lithography^35,38^ and have applied widely in the context of plasmonic hydrogen sensors.^6,18,20,28,35,38−45^ The particle dimensions are determined by the mask and their composition by the amount of evaporated alloy constituents, which can be accurately controlled by the thickness of the subsequently evaporated layers (Figure 1a–g).^20,35,38^ Here we note that it is important to consider the order of alloy constituent evaporation due to the continuous buildup of material around the rim of the holes in the mask, which results in a continuous shrinking of the diameter of the hole, and thus of the growing nanostructure, where the rate of shrinking depends on the evaporated material.^46^

**Figure 2:**
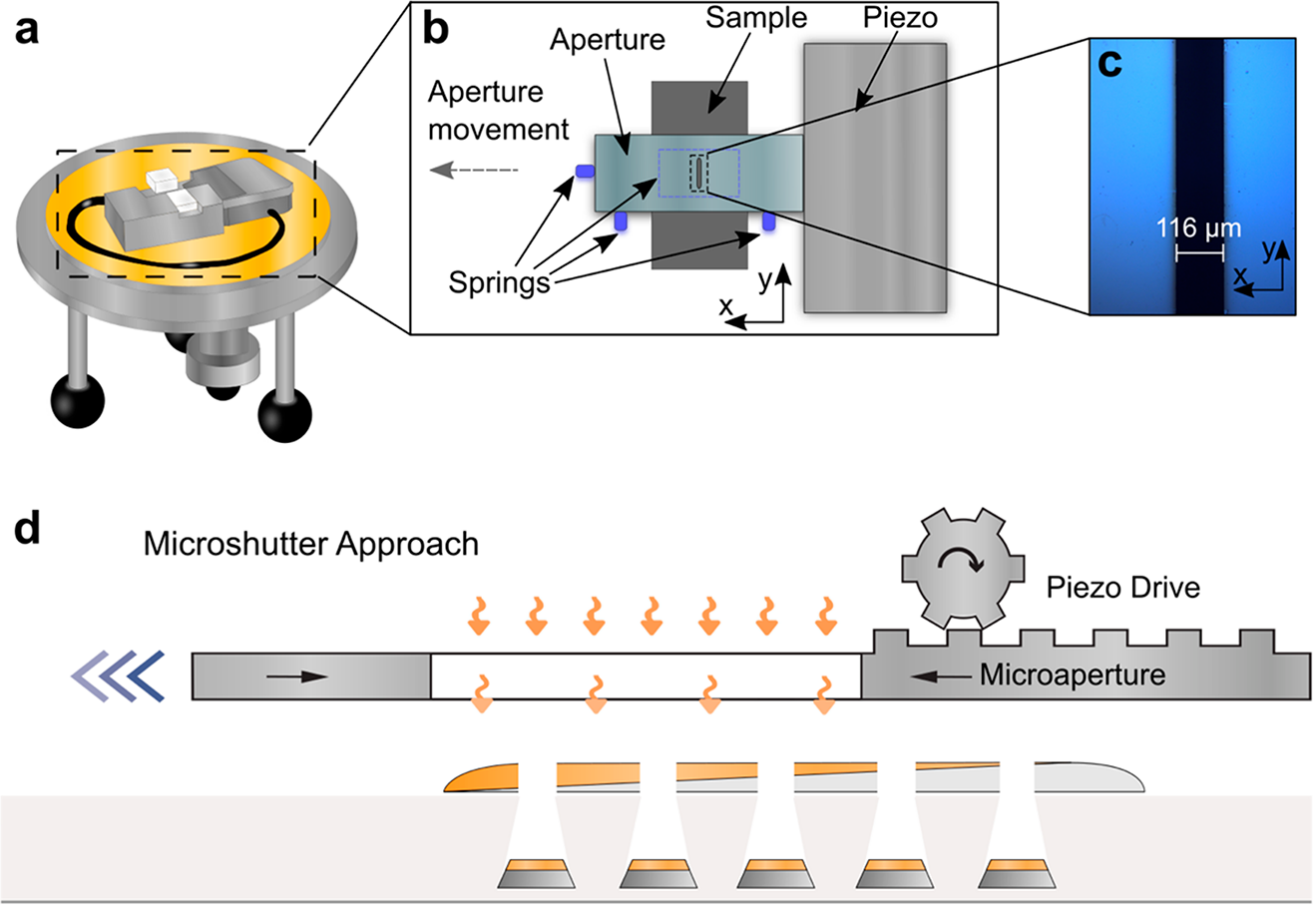
Microshutter and its working principle. (a) Schematic of the complete microshutter device (outlined with a dashed box) mounted on a standard PVD system lid. (b) Schematic of the operational parts of the microshutter. Springs are used both from the sides and from the top (indicated by a dashed outline) to push the microaperture against the piezo and the sample, respectively, to ensure correct positioning of the sample relative to the aperture. (c) Bright-field optical microscope image of a 116 μm microaperture. The silicon membrane appears as blue-tinted, and the etched opening as a black rectangle. The roughness of the edge is ∼1 μm. During operation, the aperture is moved across the underlaying sample in the *x*-direction. Total length of the aperture (not shown) in the *y*-direction is ∼5 mm. (d) Schematic depiction of alloy gradient nanofabrication using the microshutter on the example of two different metals that are subsequently evaporated through a nanolithography mask using the moving microaperture.

So far, however, this approach has been limited to alloy formation in nanostructure arrays with *identical* composition across the entire sample. To overcome this limitation and as the key advance introduced in this work, we present a microshutter device that can be mounted onto a standard PVD system, which in our case is a Lesker PVD 225 (Figure 2a–c). As the key components, the microshutter device is composed of a piezoelectric actuator that, for the samples discussed in this work, drives a 116 μm wide microaperture (Figure 2b,c, details in Methods for its fabrication, and we note that smaller or larger apertures can be used to adjust the spatial resolution to the required level) across the sample surface during evaporation of a specific element. Hence, as the aperture moves across the sample, it defines both the specific area onto which deposition takes place and the thickness of the grown film (Figure 2d). In this way, by subsequently evaporating multiple layers of different metals with tailored thickness through the narrow microaperture, alloy nanoparticles with different compositions can be produced with spatial resolution that is dictated by the minimal step size of the piezo driver and the dimensions of the aperture. Since this minimal step size is 400 nm in our case, we can principally produce two particles with different alloy composition along the evaporated material gradient if they are 400 nm or more apart.

A second important aspect required to guarantee high accuracy of the alloy nanoparticle composition in a specific spot is the precise alignment between the sample and the aperture. To achieve this, the sample substrate is diced with micrometer precision to fit into a dedicated holder with an alignment accuracy of ±1 μm in the *x*-direction (the direction in which the gradient is formed) and an angular accuracy of 0.007 deg (Figure 2b, details in Methods). Side- and top-mounted springs (Figure 2b) ensure that the aperture is kept in place during the entire process. A third key aspect is that the sample, once aligned, is firmly fixed within the slot, eliminating any movement during evaporation. This is guaranteed by plastic clamps (not shown in the drawings in Figure 2b).

**Figure 3:**
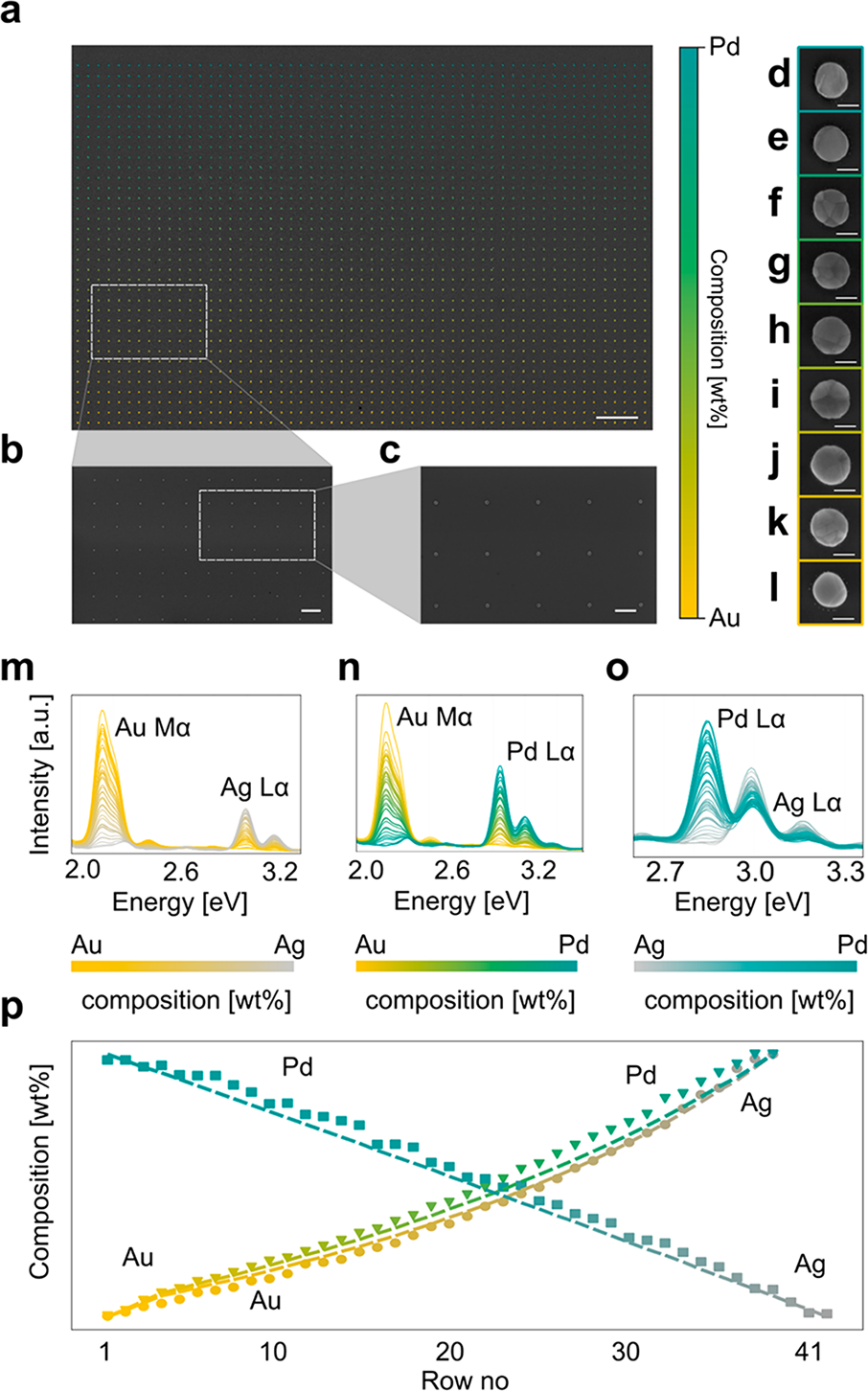
SEM micrographs and SEM-EDX composition analysis of single particles in a compositional gradient array. (a) Low-magnification SEM overview image of a AuPd alloy nanodisk array with the compositional gradient indicated by the color code superimposed to the image. The composition varies in discrete row-by-row steps from 100% Pd (top) to 100% Au (bottom). The entire array consists of 38 rows, each with 88 particles of a single, well-defined composition. Scale bar is 10 μm. (b, c) Higher magnification SEM micrographs of selected areas from a. Scale bar is 2 μm in (b) and 1 μm in (c). High-magnification SEM micrographs of individual nanodisks of selected AuPd alloy compositions in Au wt %: 0 (d), 7 (e), 26 (f), 42 (g), 56 (h), 68 (i), 79 (j), 89 (k), 100 (l). Scale bar is 100 nm. Corresponding micrographs for AuAg and AgPd can be found in Figure S1. SEM-EDX spectra for each composition for the three binary alloys AuAg (m), AuPd (n), and AgPd (o). Each line represents the spectra taken from a single particle of each composition, and the color of the line represents the bimetallic composition of that particle in accordance with the color bar below each figure. The increase in the intensity of one metal’s peak at the expense of the second metal’s peak across the sample corroborates the anticipated systematic change in alloy composition across the gradient array. (p) The composition extracted from the EDX spectra in (m)–(o) for AuAg (circles), AuPd (triangles), and AgPd (squares) plotted as a function of position in the array, together with the expected nominal composition values (dashed lines). The mean deviation from the nominal target values is 1.7 wt % for AuAg, 2.8 wt % for AuPd, and 3.4 wt % for AgPd. The standard deviation of the composition for repeated SEM-EDX measurements on the same particle was 1.5 wt %. The nonlinear dependence of composition on position in the array is a consequence of the mask hole-closing effect in combination with the different atomic weights of the alloy constituents, as discussed in the main text.

To demonstrate and evaluate the capabilities of the microshutter, we fabricated and characterized AuAg, AuPd, and AgPd binary alloy nanodisk arrays on oxidized Si substrates (Figure 3a–l and Figure S1). We chose these three specific metals due to the excellent plasmonic properties of Au and Ag, and Pd due to its relevance in catalysis and gas sensing.^18,47−50^ To prepare the evaporation masks, we used electron-beam lithography (EBL) and designed regular arrays with a lattice constant of 2.5 μm. These arrays comprised 38 (AuAg, AuPd) or 43 (AgPd) rows of particles, each of which ultimately represents a specific alloy composition, where each row contains 88 particles. This corresponds to a total of 3344 and 3784 particles in an array, respectively. The nominal disk-shaped particle diameter was set to 160 nm at the base, and we aimed at a total height of 30 nm after the evaporation of both elements. For all three alloy systems, we implemented a gradient composition profile that starts with 100% of metal A in the top row of the array, from which the composition is gradually shifted by the equivalent of 7 Å of deposited material per row to 100% of metal B in the bottom row (Figure 3b). To induce the initial alloy formation after the metal evaporation step, the samples were annealed at 500 °C for at least 15 h in 2% H_2_ in Ar carrier gas, resulting in disklike structures typically comprised of a single or several grains in terms of their morphology (Figure 3d–l).

To evaluate the specific alloy compositions obtained at each row of particles and compare it with the nominal one, we carried out energy-dispersive X-ray spectroscopy (EDX) measurements using a scanning electron microscope (SEM) on at least 38 particles across all compositions for each alloy system (Figure 3m–o; see Methods for details). Plotting the obtained alloy composition values together with the targeted nominal values reveals very good agreement and thus corroborates the accuracy of our method (Figure 3p). Here, we also note that due to the truncated conical shape of the particles during metal evaporation because of the hole-closing effect in the mask (cf. Figure 1a) and the different atomic weight of the alloy constituents, both the nominal and measured composition profile are slightly curved rather than a straight line and slightly different for different materials, since they exhibit a slightly different hole-closing rate and atomic masses. This is because the alloy composition is calculated as a thickness of deposited material rather than in wt % that is plotted in the figure and because these two parameters are not perfectly linearly related.

Finally, we note an average deviation from the nominal composition of 1.7 wt % for AuAg, 2.8 wt % for AuPd, and 3.4 wt % for AgPd. The main reason for these deviations and the fact that they are different for the different alloy systems is that the evaporation rate from different metal sources is different and not constant over time (in our specific system it fluctuates up to a few percent). However, the programming of the aperture movement across the sample assumes a constant evaporation rate (details in SI Section S4: Evaporation rate analysis).

**Figure 4:**
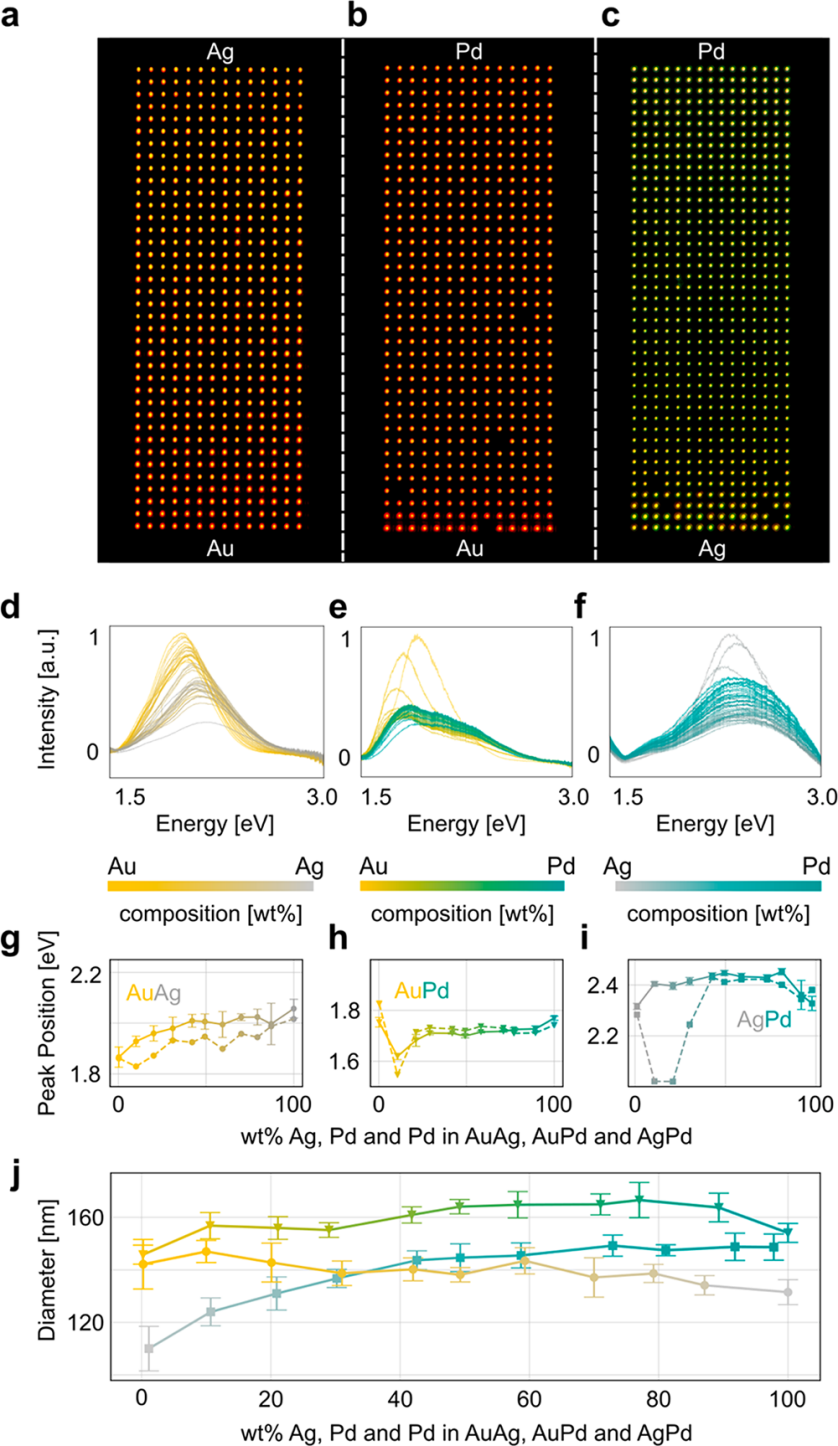
Composition-dependent single particle plasmonic properties of AuAg, AuPd, and AgPd alloys. Dark-field scattering microscopy images of AuAg (a), AuPd (b), and AgPd (c) alloy gradient arrays comprised of nanodisks with a nominal diameter of 160 nm and height of 30 nm. Correspondingly measured normalized dark-field scattering spectra from a single nanodisk for each alloy composition for AuAg (d), AuPd (e), and AgPd (f). The mean LSPR peak position for five nanodisks of identical alloy composition taken in roughly 10 wt % steps (solid lines) for AuAg (g), AuPd (h), and AgPd (i) compared to the corresponding values obtained from FDTD simulations of a single nanodisk on oxidized Si (dashed lines). The disk dimensions and compositions used in the FDTD simulations for each alloy composition are obtained by SEM and EDX measurements of the corresponding experimental particles, respectively. (j) Size distribution of 10 alloy particles for each composition presented in parts (g)–(i) as determined by SEM image analysis. Error bars indicate one standard deviation. Note the distinct composition dependence, which is important to be aware of when simulating the corresponding optical response of alloy nanoparticles with different composition nanofabricated by using the layer-by-layer and thermal annealing approach.

Having established the microshutter-based fabrication of alloy nanoparticle arrays with composition control at the level of the single particle, we analyzed their optical properties using dark-field scattering microscopy as a first evaluation of their composition-dependent properties. This method is widely used for the characterization of individual metal nanoparticles and their optical/plasmonic properties.^11^ Corresponding dark-field scattering images of a representative AuAg, AuPd, and AgPd alloy nanoparticle array comprised of nanodisks with 160 nm diameter and 30 nm nominal thickness with compositional gradient reveal a systematically varying scattering intensity, as well as color of the particles, which indicates spectral shifts of the localized surface resonance (LSPR) as a function of alloy composition (Figure 4a–c), in good agreement with measurements on nanoparticle ensembles,^20^ and corroborates the presence of a compositional gradient across the array.

To further verify and quantify these apparent trends, we first plot representative scattering spectra of single particles measured along the composition gradient for each alloy system (Figure 4d–f) and then extracted the photon energy of the LSPR peak for 11 selected alloy compositions across the entire range in about 10 wt % steps, including the two pure alloy constituents. The obtained data points were then compared to the corresponding values obtained from finite-difference time-domain (FDTD) electrodynamic simulations that used the dielectric functions from Rahm *etal*.^20^ as input (Figure 4g and Figure S2), together with nanoparticle dimensions derived from SEM images of nanoparticles with corresponding alloy compositions (Figure 4h). This alloy-specific matching of particle geometry between experiment and simulations is important since the particles attain slightly different aspect ratios after the annealing step, which induces the alloy formation, due to alloy-specific interfacial energies^20^ and since the actual diameters obtained generally slightly deviate from the nominal one (which in our case was 160 nm).

A second aspect of importance for the FDTD simulations is the thickness of thermal SiO_2_ on the silicon wafer substrate used for the experiments. Therefore, for each sample we accurately measured it using ellipsometry and used the obtained value in the corresponding FDTD model.

By taking these factors into account, we find excellent agreement between the simulated and experimental results for the AuPd and AuAg systems. Furthermore, the almost linear variation of the LSPR peak position with composition in the AuAg system is in good agreement with ensemble extinction measurements of the same system.^20,51^ On the same note, we mention that the different trend observed for the AuPd system compared to our earlier work is the consequence of different particle dimensions in combination with a thermal SiO_2_ layer present on the Si substrate (discussed below), which leads to a different spectral position of the LSPR at which the complex dielectric functions of the alloys are substantially different.^20^

Now turning to the AgPd system, we find a generally very reasonable agreement between experiment and theory but also quite significant deviations for the particles with a Ag content between 70 and 90 wt % (Figure 4i). As the main reasons for these deviations, we identify that the dielectric functions in the Ag-rich regime of the AgPd alloy system are somewhat inaccurate. The latter is in line with corresponding results of Rahm *etal*., where the calculated plasmonic response for the AgPd system showed the largest deviation from the experimental results.^20^

Finally, we note that the reason for the apparently different response for the pure elements Ag, Au, and Pd in the experiments is a consequence of different thermal SiO_2_ thicknesses on the substrate of the different samples, which leads to slightly different peak positions due to different interference between light scattered/reflected from the different interfaces in the system (Figure S3).

In the last part of this work, we will demonstrate the usefulness of nanostructured surfaces made using the microshutter for the screening of the impact of alloy composition on the single nanoparticle function. As our model system we use the AuPd alloy system, which we have widely used and characterized in the context of plasmonic hydrogen sensors.^6,35,38,44,45^ Such sensors are based on the principle that hydrogen absorption into interstitial sites of metallic hosts, such as Pd and its alloys but also other systems like Ni,^52^ Mg,^53^ Y,^54^ Hf,^55,56^ and Ta,^55^ and their alloys with Pd,^57^ induces distinct changes in their electronic structure, and thus optical response in general and LSPR in particular. Importantly, these changes are proportional to the hydrogen concentration/partial pressure in their surroundings.^58−60^ From the application perspective, such sensors are attractive since their optical signal transducing mechanism generates no sparks and since the entire sensor, in principle, can be miniaturized down to a single nanoparticle.^10,61−64^ The former aspect is especially important since hydrogen–oxygen mixtures are highly flammable above the 4% H_2_ lower explosive limit.

Mechanistically, when Pd and its alloys are exposed to hydrogen gas, the H_2_ molecule dissociates on the surface at ambient conditions and the formed H-species diffuse into the host, where they occupy interstitial lattice sites.^65^ The reason for alloying Pd with other metals in this context is mainly twofold. First, in pure Pd the absorption of hydrogen gives rise to a first-order phase transformation from the hydrogen-poor solid solution (α-phase) to the hydrogen-rich hydride (β-phase), which is accompanied by strain-induced hysteresis between hydride formation and decomposition that is characterized by a distinct “plateau” in pressure–composition isotherms.^58,65,66^ This hysteresis leads to ambiguous sensor readout and dramatically reduced sensor accuracy.^45^ Importantly, however, introducing elements with different lattice parameters into the Pd lattice, *e.g.*, alloying with other metals such as Au, Ag, Ni, or Cu, is an effective way to eliminate hysteresis through prestraining of the lattice and a reduction of the critical temperature, thereby significantly improving sensor function.^18,38,67,68^ As the second aspect, alloying also constitutes a pathway to optimize the kinetic barriers that dictate the rate at which hydrogen is absorbed into the lattice and thus the response time of hydrogen sensors.^45^

As the first experiment in this context with a microshutter-made sample, we used a AuPd alloy gradient system comprised of 3344 nanoparticles organized in 38 rows of 88 particles and with alloy composition varied by 1–5 wt % from row to row (exact value depends on the position in the array due to the hole-closing effect), which we exposed to a 200 mbar pulse of H_2_ from vacuum at 303 K, using a setup reported earlier^61^ (see also Methods for details).

**Figure 5:**
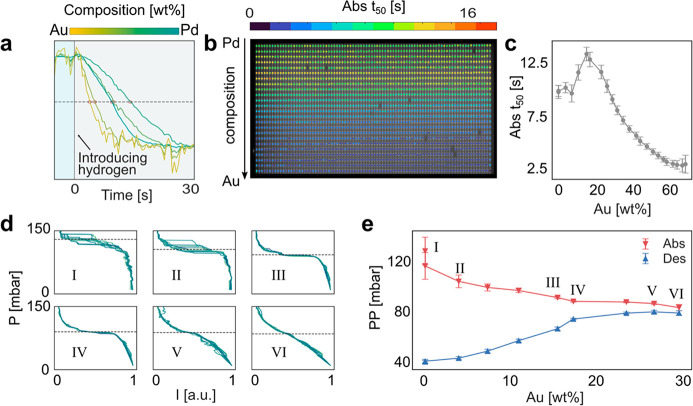
Hydrogen sorption kinetics and isotherm measurements of single AuPd alloy nanoparticles. (a) Normalized dark-field scattering intensity time traces for single particles of five distinct compositions (from left to right: 58, 44, 0, 29, and 15 wt % Au, which represents rows 20, 15, 0 10, and 6 from the top in (b)) upon exposure to a ∼200 mbar H_2_ pulse (indicated by the blue and gray boxes) at 303 K. The time traces are color-coded according to the particles’ compositions. The individual *t*_50_ value, which is defined as the time to reach 50% signal intensity change after hydrogen exposure, is represented with a red circle in each of the time traces. (b) Dark-field scattering image of an array of AuPd alloy particles starting at 0 wt % Au (top row) and extending to 68 wt % Au (bottom row). The image is color-coded with the extracted *t*_50_ values for each particle, as obtained from the time trace of exposure to a ∼200 mbar H_2_ pulse in (a). (c) Mean *t*_50_ values for hydrogen absorption in 88 particles of each alloy composition plotted as a function of composition, as extracted from (b). (d) Optical pressure–composition isotherms for 10 selected single nanoparticles for each alloy composition, as measured from a second AuPd alloy gradient sample at 333 K. The mean two-phase coexistence plateau pressure values from the 10 particles are indicated by a dashed line for each alloy composition. Note the decreasing spread in plateau pressures for increasing Au content in the alloy. (e) Mean plateau pressures from 88 particles for each alloy composition from 0 to 29 wt % Au for hydrogen absorption (red, downward pointing triangles) and desorption (blue, upward pointing triangles). Note the decreasing hysteresis width as well as spread between individual particles with nominally identical alloy composition for increasing Au concentration.

Using the single particle plasmonic nanoimaging approach,^61^ we simultaneously measured the drop in scattering intensity from each individual nanoparticle in the array induced by the hydrogen pulse as a function of time and plot selected corresponding time traces for single particles with different alloy composition in Figure 5a. Evidently, increasing the Au content significantly alters the absorption of hydrogen in the single particles.

To now quantify the response from all particles in the array, we define the response time, *t*_50_, as the time to reach 50% scattering intensity change (Figure 5a) and plot the obtained values as a color-coded dark-field scattering microscopy image of the array ranging from pure Pd (top row, Figure 5b) to 68 wt % Au in Pd (bottom row, Figure 5b). Here we note that for higher Au concentrations no hydrogen is absorbed and therefore no scattering intensity change is detected. This analysis reveals a distinct composition dependence of the response time, as well as that different individual particles nominally localized in a row with identical composition exhibit slightly different response, in good agreement with our earlier studies of single pure Pd nanoparticles, in which we identified morphological differences as the cause.^61,62^ To further analyze the obtained data, we plot the obtained *t*_50_ values as a function of alloy composition, which reveals an initial increase in absorption time for low Au concentrations compared to pure Pd, followed by continuous acceleration of the response for Au concentrations > 15 wt % (Figure 5c). As an interesting detail, we notice that the error bars on the data points for each alloy composition are systematically reduced for increasing Au concentration (the increase observed at the highest Au concentrations is a consequence of the very small optical contrast generated upon hydrogen absorption in these particles). Since the number of single particles considered is the same for each alloy (88) and all particles were measured simultaneously, this is a clear indication of “particle individuality” being correlated with alloy composition. We speculate that the origin of this effect is to be sought in the morphology of the particles, which we have previously shown to have a profound effect on the hydrogen sorption kinetics of pure Pd nanoparticles.^61^ Taken all together, these results hint at a trend in which a higher Au content produces nanoparticles with more homogeneous grain structure upon annealing during alloy formation. This argument is also strengthened by the lower melting and recrystallization temperatures of Au compared to Pd. As the final point, we highlight here that this observation, *i*.*e*., that it can be made, is a direct consequence of the design of our sample enabled by the microshutter and our ability to measure many single particles for many different alloy compositions in a single experiment. In other words, it would be very difficult, if not impossible, to observe this in separate experiments for each alloy composition due to inevitable experiment-to-experiment errors and corresponding uncertainty in the data.

As a second experiment executed on a different AuPd sample to demonstrate the reproducibility of the microshutter-based nanofabrication, we addressed the hysteretic properties of the system as a function of alloy composition. Specifically, we measured optical pressure–composition isotherms for Au concentrations up to 30 wt % Au at 1–5 wt % increments from row to row (exact value depends on the position in the array due to the hole-closing effect and the different atomic weight of Au and Pd) and 88 single particles for each concentration and plot the extracted two-phase coexistence plateau pressures for both hydrogen absorption and desorption as a function of alloy composition (Figure 5d,e). As the main result, we find a systematic reduction of the hysteresis gap, in very good agreement with our previous studies.^44,45^ Also here, we note the distinct reduction in the spread of the plateau pressures identified for the individual nanoparticles of identical composition for increasing Au concentration. This can again be understood on the basis of the impact of particle microstructure in terms of abundance of crystallites since it is to be expected that the strain-driven correlation between hysteresis width and number of crystallites identified for pure Pd^62^ is reduced as a consequence of the lattice prestraining effect when substituting Pd atoms with (larger) Au atoms in the system.

## Conclusions

In conclusion, we have reported the implementation of a piezo-controlled microshutter device compatible with a standard PVD system, which enables spatially highly resolved thin film evaporation in lithography-based fabrication of nanostructured surfaces with compositional gradients. As demonstrated on the examples of AuAg, AuPd, and AgPd, using electron beam lithography this makes it possible to craft arrays composed of binary alloy nanoparticles, whose composition is controlled at the 1–5 wt % level and with spatial resolution down to 400 nm, which corresponds to the minimal step size of the presently used piezo controller. In other words, our method enables the accurate composition control of single nanoparticles in an array, combined with the accurate nanostructure size and shape control offered by nanolithography in general and by electron beam lithography in particular. This is an important advance because the accurate experimental scrutiny of the impact of subtle changes in alloy composition on nanomaterial function is challenging due to the large parameter space that must be considered, *i*.*e*., nanostructure size, geometry, chemical composition, and structural atomic-level differences between individuals, which leads to unrealistically large sample sets if statistically relevant and systematic data are to be obtained.

As the second key result of this work and in response to this challenge, we have demonstrated that using arrays of nanoparticles with compositional gradients, it becomes possible to address this large parameter space in a single experiment using plasmonic nanospectroscopy and imaging. Specifically, in a first set of experiments, we have characterized the plasmonic response of single AuAg, AuPd, and AgPd alloy nanoparticles as a function of composition and found very good agreement in terms of spectral position of the LSPR with corresponding FDTD simulations using the complex dielectric functions from Rahm *etal*.^20^ as input. In a second set of experiments, we investigated PdAu alloy gradient arrays nanofabricated by using the microshutter device for their hydrogen sorption properties. Here, we found distinctly composition-dependent hydrogen sorption kinetics and hysteresis, in good agreement with the common understanding of the effect of alloying Pd with Au on hydrogen sorption and hydride formation. As an additional interesting observation, we found an alloy composition-dependent contribution of the single nanoparticle response to the ensemble average of 88 particles for each alloy composition, which revealed itself as a decreasing spread in both single-particle specific hydrogen absorption time and hysteresis width in optical pressure–composition isotherms. As the reason, we identified a decreasing significance of lattice strain upon (de)hydrogenation due to the increasing lattice prestraining by Au upon increasing Au concentration in the alloy. These findings corroborate the significance of and add value to alloy composition screening in single experiments with single nanoparticle resolution, since they likely could not have been obtained in a large number of subsequent experiments on individual samples representing a simple alloy composition each. In a wider perspective, our work advertises the concept of microshutters for the PVD of thin films, as well as for nanostructuring based on nanolithography, such as electron beam lithography, colloidal lithography, or photolithography, to achieve (nanostructured) surfaces with composition and/or thickness gradients with nanoscale spatial resolution.

## Methods

### Microshutter

The microshutter mechanism principally consists of three distinct parts: (i) an exchangeable microaperture, (ii) a drive that moves the aperture across the sample, and (iii) a slot for the sample (Figure 2b). Springs were used in both the vertical and the horizontal plane to push the aperture against the piezo platform and the sample beneath the aperture, respectively. To push the aperture across the sample, we use an *x*-axis piezoelectric actuator (PX 400 SG Piezostage VAC) with a controller by Piezosystem Jena GmbH. This piezo positioner has a travel range up to 300 μm and can operate in vacuum. The whole setup is mounted on a standard evaporator lid with a vacuum-efficient inlet port for electrical wiring (Figure 2a).

The apertures were fabricated from 4 in. Si(100) wafers with a thickness of 500 μm. Marks for dicing were made with standard laser lithography using a DWL 2000 Heidelberg Instruments laser writer (HMDS, LOR3A 3000 rpm-1500acc-60s, HP 180C 1 min, S1813 3000 rpm-1500acc-60s, HP 110C 1 min, DWL 15-100-100; development: MF319 1 min) and e-beam evaporation of CrAu 100A–800A using PVD 225 (Kurt J. Lesker). Lift-off of the mask was done overnight in acetone, IPA, and H_2_O with a final plasma cleaning (dry etch RIE, Plasma-Therm, O_2_ plasma 100 W, 2 min). A 500 nm layer of Al was sputtered on the whole surface (FHR MS 150 sputter). Standard laser lithography was used to define the aperture opening (HMDS, S1813 4000 rpm-2000acc-60s, HP 110C 1 min; development: MF319 1 min 15 s) and etched with Al etching (10 min in Al etching bath with heating (starting from RT), with agitation) and a Si etching Bosh process (1500 cycles, about 7 h 45 min). Finally, the aperture was cut out from the wafer (DAD3350/Disco dicing saw, blade *k*010-600-JXS 250um) and cleaned with Al etching (about 10 min with heating starting from RT, with agitation). With this method a minimum aperture width of 20 μm can be achieved, and the roughness of the edge is about 1 μm (Figure 2c). Here we used an aperture with an opening width of 116 μm and a length of ∼5 mm.

### Sample Preparation

The samples were prepared on either a 4 in. or a 6 in. Si(100) wafer of 500 μm thickness. All samples have a dry oxide layer (80–130 nm depending on the sample, Centrotherm furnace). Marks for dicing were made in the same way as those for the aperture. The samples were diced (DAD3350/Disco dicing saw, blade *k*010-600-JXS 250um, surface protection: S1813 1 min HP110C) to precisely fit into the fixed sample slot of the microshutter. To make sure the edges of the samples were straight and not curved (due to the edge of the blade), three layers of dicing tape (total of 0.225 mm) were used to support the wafer, and the blade height was set to 0.05 mm. EBL (RAITH EBPG 5200) was used to define the nanoparticle mask (MMA 8.5 MMA-EL6 4000-2000-1 min, HP 180C 5 min, PMMA 950-A2 4000-2000-1 min, HP 180C 5 min; development: 3:1 MBI:IPA 1 min, rinse in IPA, blow dry) with 3 s of O_2_ plasma cleaning (50 W, dry etch RIE, Plasma-Therm) before metallization. In this work, we used an e-beam thin film evaporator (Kurt J. Lesker PVD 225) for metal deposition. It has a base pressure of 3 × 10^–8^ Torr and various pockets (Ti, Al, Cr, Au, Ge, Pd, Ni, Pt, Ag, Cu, V). Lift-off of the mask occurred overnight in acetone, IPA, and H_2_O. Samples were annealed in a flow furnace (Nabertherm R50/250/12) under 2% H_2_ in Ar (300 mL min^–1^) at 500 °C for 15 h after fabrication to induce alloy formation. Some samples were annealed again before measurements if they showed signs of oxidation or segregation (due to storage under ambient conditions).

### Energy-Dispersive X-ray Spectroscopy Measurements

The EDX measurements were performed on a JEOL 7800F Prime scanning electron microscope equipped with an X-Max N 80 detector from Oxford Instruments. The instrument was operated at 10 kV, and a working distance of a maximum of 10 mm was used. The spectra were acquired for 80 s with an aperture of 110 μm. The spectra were corrected and analyzed with Aztec software, version 5.0.

The elemental EDS mapping and STEM images were acquired with an FEI Titan 80-300 (300 kV) equipped with an INCA X-sight detector (Oxford Instruments). The sample holder was tilted about 20° toward the detector. The X-ray spectra were background corrected, and peaks fit in mixed mode (standard peaks were used for elemental peaks in the standards list and theoretical Gaussians for any, if present, peaks not in the standards list) using FEI TIA version 5.12.

### Single Particle Kinetics Measurements

The samples were placed in a vacuum-tight microscope chamber (custom-made Linkam 350 V) and secured with metal clips eliminate movement during the measurement. The chamber was then aligned under an upright optical microscope (Nikon Eclipse LV100, Nikon 50× BD objective) equipped with a motorized stage (Märzhäuser), and the outlet of the stage was connected to a vacuum pump (Pfeiffer, TSU 071) via a pneumatic valve (Figure S4). The system was initially evacuated to ∼1 μbar pressure, and mass flow controllers (Bronkhorst) were used to build up pressurized 100% H_2_ gas (6.0 purity) in the tubes leading to the closed chamber prior to measurements. The latter was to make sure that the inlet of H_2_ was fast (a pulse rather than a continuous flow). During the hydrogen absorption measurements, the samples were measured for 5 min in vacuum before the gas inlet was opened to let in at least ∼150 to 200 mbar H_2_. The system was then allowed to saturate for 5 min, after which the pneumatic valve was opened to pump the chamber back to the initial ∼1 μbar pressure. For the entire duration of the experiment, the image of the nanodisk array was captured with a thermoelectrically cooled electron multiplying charge coupled device (EMCCD) camera (Andor iXon Ultra 888). The scan rate for image acquisition was 2 frames per second. For every frame, the intensity for each particle was then obtained from the image as the sum of the brightest pixel and its four nearest neighbor pixels in the diffraction-limited spot of light from that particle. As has been previously shown,^61,62^ the intensity change is proportional to the hydrogen concentration in the Pd-containing particle.

### Single Particle Spectroscopic Measurements

The spectroscopic measurements were performed with the same microscope as the kinetic measurements, but this was now instead connected to a spectrometer (Andor Shamrock SR-303i-B). After a column of nanodisks had been aligned with the spectrometer slit (500 μm), the light scattered from the particles was dispersed onto a grating (150 lines/mm, blaze wavelength 800 nm), after which the resulting spectrum was obtained by a thermoelectrically cooled CCD camera (Andor Newton 920). By aligning an entire column with the spectrometer slit, the spectra of all 38 (43 for the PdAg sample) compositions could be measured simultaneously. The illumination source of the microscope was a 50 W halogen lamp (Nikon LV-HL50W). Normalized scattering spectra, *I*_sc_(λ), from individual nanodisks were defined as , where *I*_c_(λ) is the collected (raw) spectra from a single nanodisk, *I*_b_(λ) is the background signal (dark area without particle), and *I*_l_(λ) is the signal collected from the diffuse white certified reflectance standard (Labsphere). The latter is used to correct the signal in the lamp spectrum. The exposure time for each spectrum was, depending on the sample, 5–15 s. The obtained single-particle scattering spectra were fitted with a polynomial function (degree 5, ±50 nm from the maximum intensity) to find the plasmonic peak position.

### FDTD Simulations

Ansys-Lumericals FDTD Solution version 8.26.2779 was used to calculate the backscattering cross section of the nanodisks. The simulated geometry was composed of a single tapered cylinder with rounded edges placed on an oxidized Si substrate. The relevant geometries for each alloy system, in particular, the particle sizes and oxide thicknesses, were derived from SEM images and ellipsometry measurements, respectively. The dielectric functions for the alloys are taken from Rahm *etal*., and the Si and SiO_2_ was used out of Ansys-Lumerical’s database originating from Palik *etal*.^20,69^ The source used was a total-field/scattered-field source with a linearly polarized plane wave.

### Ellipsometry

Measurements were done with a variable-angle multiwavelength spectroscopic ellipsometer (Woollam M2000), equipped with a quartz tungsten halogen lamp and with a Si CCD parallel detector of 245 to 1000 nm spectral range (470 discrete wavelength intervals). The thicknesses of SiO_2_ films were derived by fitting the data obtained at incidence angles of 65°, 70°, and 75° to a layered model structure of a SiO_2_ layer of variable thickness on top of 1 mm thick silicon.

## Supporting Information Available

High-magnification SEM micrographs of individual AgAu and AgPd alloy nanodisks; FDTD-calculated scattering cross sections of AuPd, AuAg, and AgPd alloy nanodisks; FDTD-calculated scattering cross section of a Pd nanodisk with dimensions of the nanodisks obtained by SEM imaging their experimental counterparts; schematic overview of the experimental setup used for hydrogen sorption kinetics experiments; evaporation rate analysis (PDF)
