# Supplementary material for: A Microshutter for the Nanofabrication of Plasmonic Metal Alloys with Single Nanoparticle Composition Control
Source: ACS Nano. 2023 Aug 3;17(16):15978–88. doi: 10.1021/acsnano.3c04147 (PMC10448753; doi:10.1021/acsnano.3c04147)
Supplement: Supplementary file 1 — nn3c04147_si_001.pdf [file nn3c04147_si_001.pdf]

Supporting Information

for

A Microshutter for the Nanofabrication of Plasmonic Metal Alloys with Single Nanoparticle Composition Control

Carl Andersson♣, Olga Serebrennikova♣, Christopher Tiburski♣, Svetlana Alekseeva, Joachim Fritzsche, Christoph Langhammer**

Department of Physics, Chalmers University of Technology, 412 96 Göteborg, Sweden

♣These authors contributed equally

*joafri@chalmers.se; clangham@chalmers.se

Section S1: SEM images of AgAu and AgPd nanoparticles

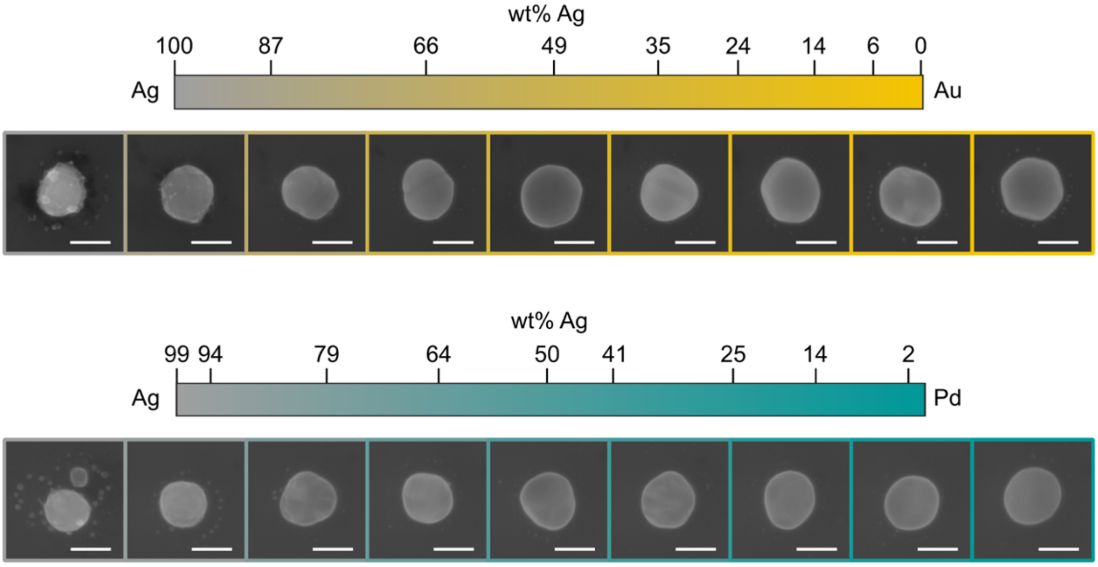

Figure S1: *High magnification SEM micrographs of individual AgAu (top row) and AgPd (bottom row) alloy nanodisks of selected compositions in wt% Ag from left to right. AuAg:100, 87, 66, 49, 35, 24, 14, 6, 0 (wt% Ag) and AgPd: 99, 94, 79, 64, 50, 41, 25, 14, 2 (wt% Ag). The observed surface inhomogeneity for the most Ag-rich systems is most likely the consequence of a certain level of surface oxidation due to residual O_2 in the evaporation chamber. This oxidation continues upon exposure to ambient conditions for the Ag-rich alloys – see Figures S2 and S3. Scale bar is 100 nm.*

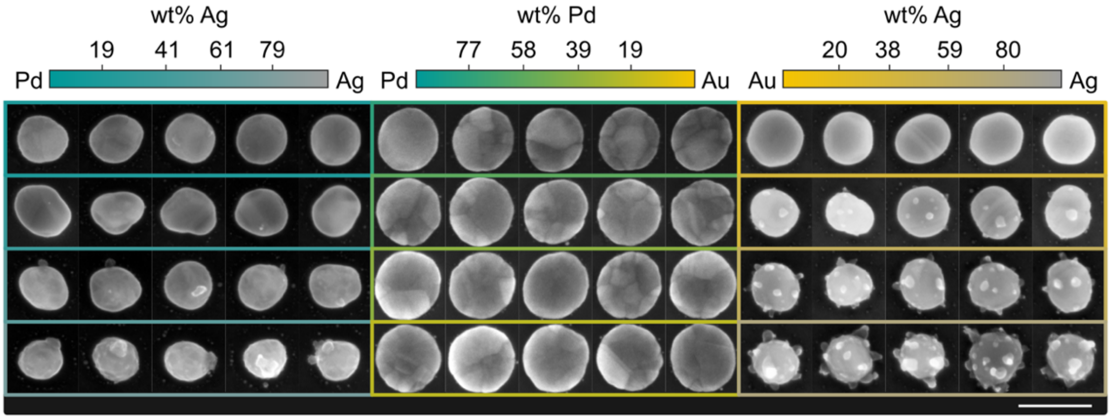

Figure S2: *High magnification SEM micrographs of individual particles of equal composition to showcase the different morphologies found within each composition. Images are arranged in three horizontal panels with AgPd (leftmost), AuPd (middle) and AuAg (rightmost). Within each panel, every row shows micrographs of 5 different nanoparticles of equal composition. The composition of each row (top to bottom) can be found from the corresponding color bar above each figure (read from left to right). These images were taken 11 months after annealing of the particles and the strikingly different morphology of the high content Ag particles can be explained by extensive oxidation over the extended time period (see also Figure S3). Scale bar is 100 nm.*

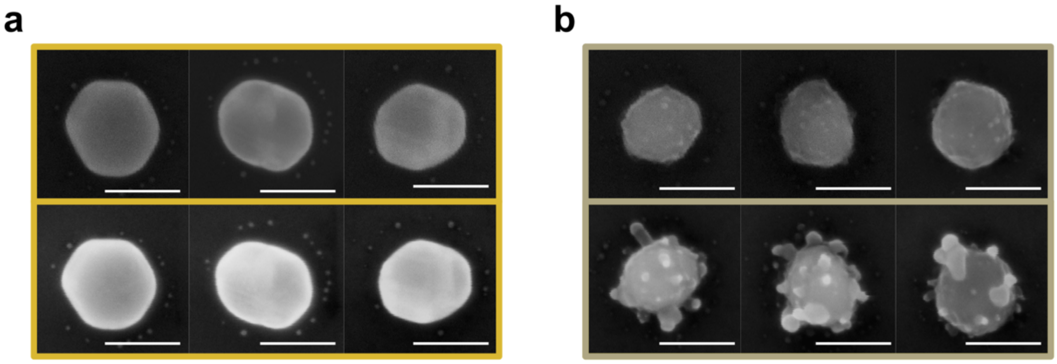

Figure S3: *High magnification SEM micrographs of individual AuAg particles showcasing the aging of high Au content (5 wt% Ag) particles (a) compared to high Ag content (85 wt% Ag) particles (b). Top row in each figure shows three particles of equal composition just after annealing and bottom row shows the same particles 11 months later after storage in ambient conditions. Notably, the Au-rich particles nicely retained their shape and morphology, whereas the Ag-rich particles have formed distinct protrusions due to oxidation. Scale bar is 100 nm.*

Section S2: Finite-difference time-domain simulations of AuPd, AuAg and AgPd nanoparticles

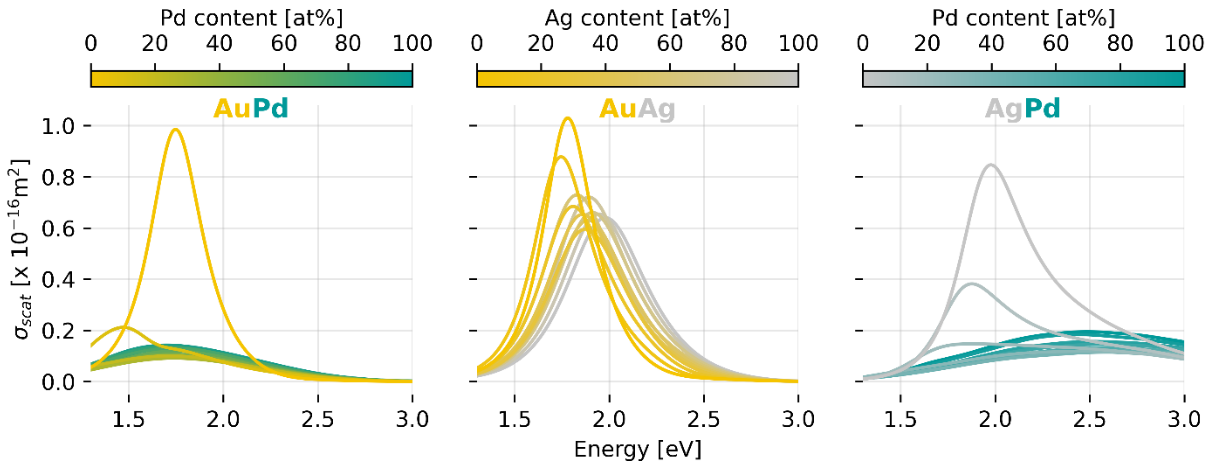

Figure S4: *FDTD calculated scattering cross-sections of AuPd, AuAg and AgPd alloy nanodisks on oxidized Si substrate. The dimensions of the nanodisks were obtained by SEM imaging their experimental counterparts (diameter after annealing given by Figure 4j and height calculated from a truncated cone model, Figure 1a, with total deposited volume together with diameter after annealing). The silicon oxide layer thickness of the substrate was determined by ellipsometry and the alloy composition of the nanodisks by SEM-EDX.*

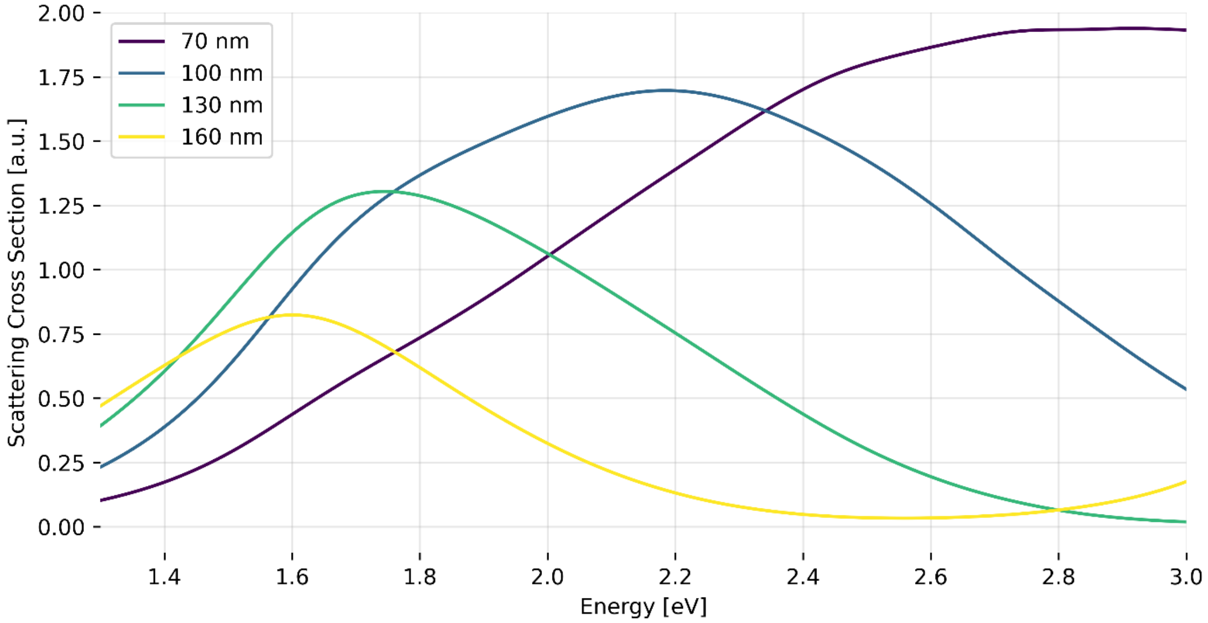

Figure S5*: FDTD calculated scattering cross section of a Pd nanodisk with a thickness of 21.9 nm and a diameter of 149.6 nm on oxidized Si substrate for different substrate SiO_2 thicknesses of 70, 100, 130, and 160 nm. Evidently, a change of the underlying SiO_2 thickness has a significant impact on the position, heigh and shape of the LSPR peak.*

Section S3: Hydrogen kinetics setup

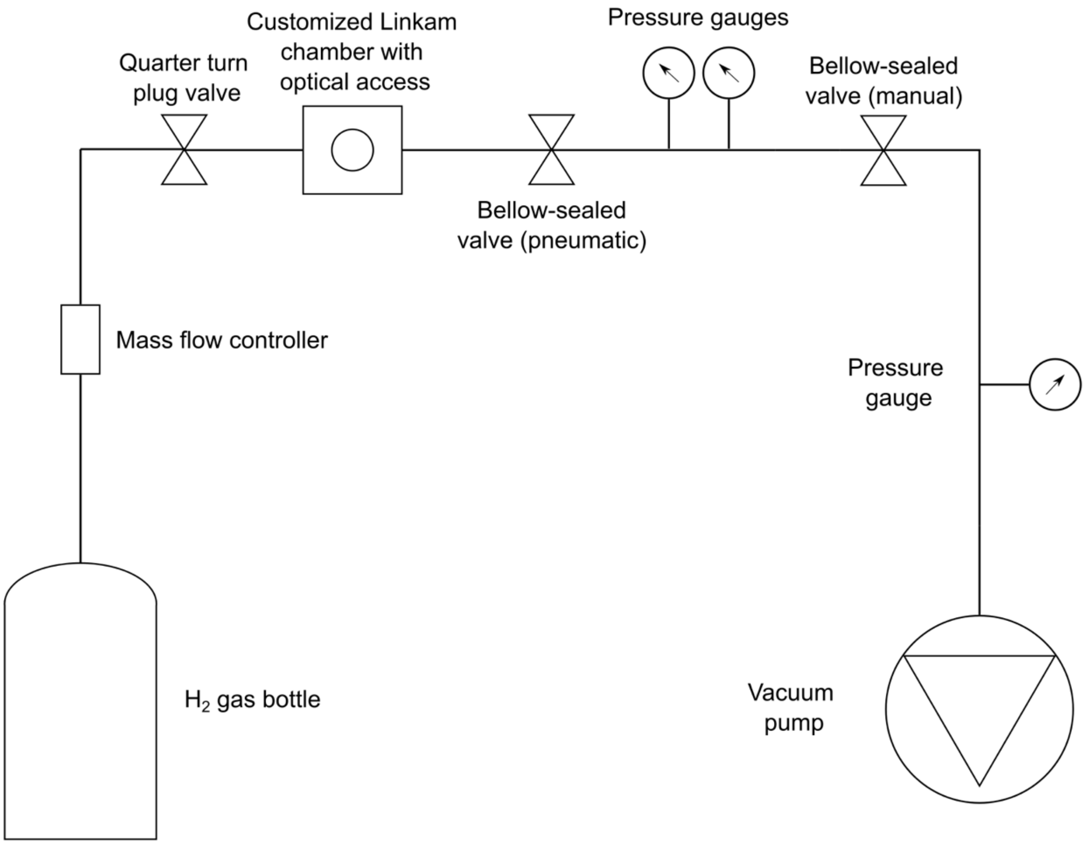

Figure S6: *Schematic overview of the experimental setup used for hydrogen sorption kinetics experiments. A customized Linkam temperature-controlled vacuum chamber was positioned on a motorized stage on an upright Nikon optical microscope. The Linkam chamber was connected on one side to a Pfeiffer Vacuum HiCube and on the other side to a mass flow controlled (Bronkhorst) hydrogen gas bottle.*

Section S4: Evaporation rate analysis

The measured fluctuation in evaporation rates around the set rate of 1 Å/s during five different evaporations for the three different metals Ag, Au and Pd is depicted in **Figure S7a**. To generate a better understanding of how the fluctuations in the deposition rate affect the composition accuracy of the alloys in a nanoparticle array, we consider the following gedankenexperiment. We assume that we fabricate a number of hypothetical thin films of Pd, Au and Ag, using *the same* deposition rate data as in **Figure S7a**. We then calculate how far off the resulting thickness would be from the nominal one, exclusively due to the deviation from the set nominal evaporation rates in **Figure S7Figure a**, as a function of deposited material film thickness in nanometers. We assume that we want to deposit a 10 nm film during 100 s of evaporation time. If the evaporation rate is constantly 1% off, then the final thickness of the film will also be 1% off the target 10 nm. The result of this gedankenexperiment is presented in **Figure S7Figure b**. We note the best accuracy is reached for Au, followed by Ag and Pd in descending order, as expected from the deposition rate data in **Figure S7Figure a**. This is in excellent agreement with the deviations we found for the three alloys in **Figure 3p** in the main text, with AuAg (1.7 wt%) showing the lowest error followed by AuPd (2.8 wt%) and AgPd (3.4 wt%). The 1.5 wt% standard deviation expected from the EDX measurements makes it difficult to draw any statistically significant conclusions, but these results at least provide good qualitative support for the material specific deposition rate variations as the mechanism behind the compositional deviations in the final alloys.

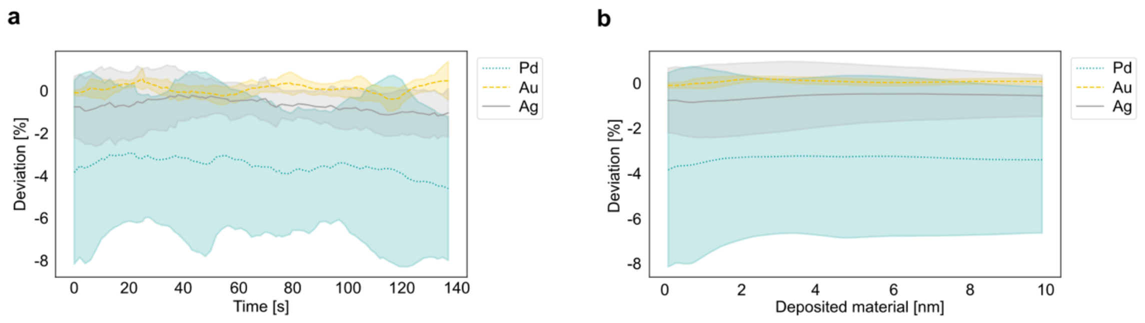

***Figure S7**: Evaporation rate variations for Pd, Au and Ag. a) Deviation in % from the target evaporation rate of 1 Å/s for Au, Ag and Pd during deposition in the Lesker PVD 225 evaporator used in this work. Mean values (lines) and one standard deviation (shaded areas) are calculated from five independent evaporations. b) Calculated representative mean error values (lines) obtained using the data from (a) from the target thickness of a hypothetical thin film as a function of the thickness of the film. Shaded areas represent one standard deviation.*
